# Mucinous Adenocarcinoma of the Lung: A Great Mimicker of Pneumonia

**DOI:** 10.7759/cureus.39343

**Published:** 2023-05-22

**Authors:** Manuel Cabrera Charleston, Daniella Lizarraga Madrigal, Asad Khan, George Eapen, Horiana Grosu

**Affiliations:** 1 Internal Medicine, Instituto Tecnológico y de Estudios Superiores de Monterrey, Monterrey, MEX; 2 Pulmonary Medicine, MD Anderson Cancer Center, Houston, USA

**Keywords:** lung adenocarcinoma, pneumonia, computed tomography, lung cancer, mucinous adenocarcinoma

## Abstract

Mucinous adenocarcinoma is a rare lung cancer that can mimic the appearance of infectious pneumonia on imaging. The present report describes the case of an 88-year-old man who presented with a cough that was not responsive to treatment. Based on chest X-ray findings consistent with pneumonia, he was treated with oral antibiotics. After the patient’s symptoms did not improve, a computed tomography scan was performed, which showed a confluent consolidation in the left lower lung and a cavitation suggestive of pneumonia. The patient was then admitted to the hospital to receive intravenous antibiotics. Although his cough continued, laboratory findings were within normal ranges and bacterial cultures were negative. He underwent two bronchoscopy procedures with bronchoalveolar lavage and was diagnosed with parainfluenza and rhinovirus/enterovirus, for which he was treated with prolonged antibiotics and steroids. His symptoms still failed to improve, and a bronchoscopy with cryobiopsy was performed, with a positive result for mucinous adenocarcinoma. This case illustrates the need to distinguish mucinous adenocarcinoma from pneumonia to improve the early diagnosis of this rare cancer and patient outcomes.

## Introduction

Lung cancer is the leading cause of cancer-related death in the world, and lung adenocarcinoma is among the most common types of lung cancer. Mucinous adenocarcinoma, formerly called mucinous bronchioalveolar carcinoma, is the rarest type of lung adenocarcinoma [1,2]. Mucinous adenocarcinoma can be difficult to differentiate from pneumonia owing to their similar radiological findings. Typical computed tomography (CT) findings indicative of mucinous adenocarcinomas, such as consolidations and opacities, can mimic those of pneumonia. We present a case in which a mucinous adenocarcinoma of the lung was initially diagnosed as pneumonia.

## Case presentation

**Figure 1:**
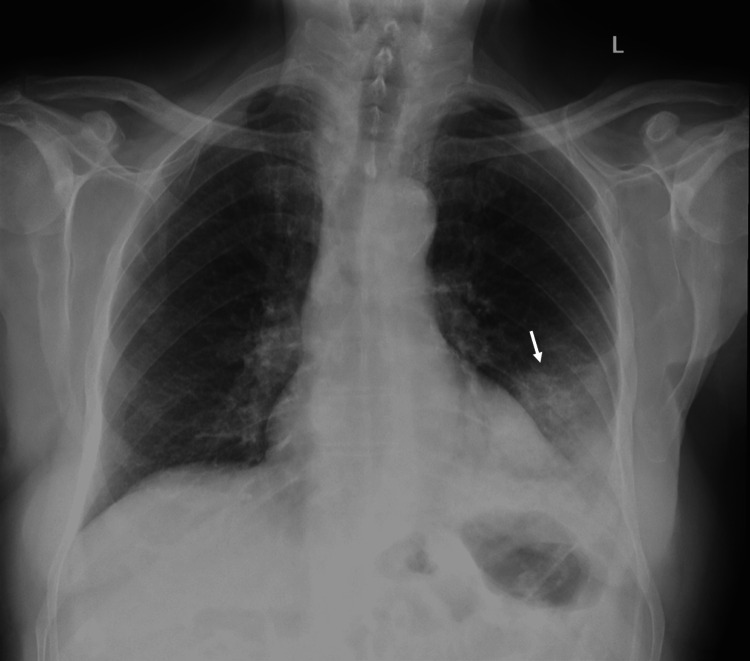
Chest X-ray showing left lower lobe pneumonia.

**Figure 2:**
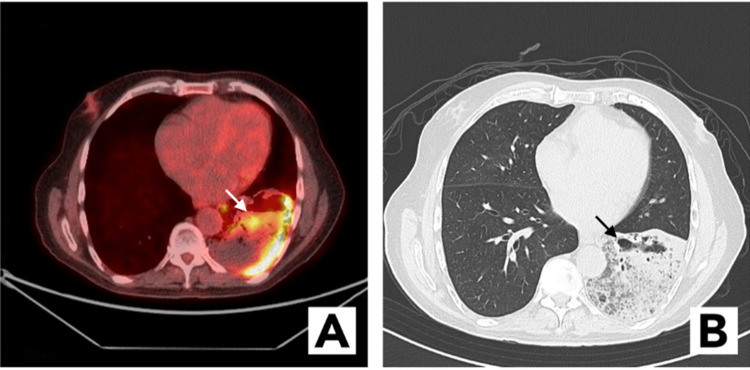
(A) Positron emission tomography-computed tomography image with fluorodeoxyglucose-avid areas in the left lower lobe. (B) Computed tomography image with large consolidative changes and ground-glass opacities in the left lower lobe.

The patient was an 88-year-old frail man who initially presented to an urgent care center with a cough. The cough was productive with clear phlegm. His cough was mostly at night and occasionally postprandial. He denied fever and chills and had no hemoptysis. He did complain of pleuritic chest pain and dyspnea on exertion. He had a history of diabetes mellitus and hypertension and was a lifelong nonsmoker. Based on chest X-ray findings showing a left lower lobe consolidation, the patient was treated with oral antibiotics for 10 days for presumed pneumonia (Figure 1). Two days after completing the antibiotic treatment, his symptoms of productive cough with clear phlegm persisted, and a CT imaging was performed, which showed a dense consolidation with a cavitary lesion in the left lower lobe. He was given a second course of antibiotics for presumed pneumonia for 14 days. In view of no improvement after 14 days of antibiotics, the patient was admitted to the hospital for treatment of left lower lobe pneumonia with intravenous antibiotics. In addition, due to the finding of cavitation, tuberculosis and aspiration pneumonia with abscess formation were in the differential. He underwent a bronchoscopy with bronchoalveolar lavage with negative cultures, including mycobacterial cultures for tuberculosis. His cough, dyspnea, and chest pain improved, and he was discharged with prolonged oral antibiotics for one month for presumed aspiration pneumonia with abscess formation. However, just a week after discharge, the patient’s cough recurred and was productive with clear phlegm. He was readmitted to the hospital for further workup. A speech and swallow evaluation with an esophagogram was performed to evaluate silent aspiration which was negative. A repeat CT scan showed a similar finding of dense consolidation with a cavitary lesion in the left lower lobe. For this, he underwent a second bronchoscopy, this time with full staging endobronchial ultrasound and biopsy of contralateral hilar, mediastinal, subcarinal, and ipsilateral mediastinal and hilar lymph nodes, and no malignancy was found. He was again treated with intravenous antibiotics and steroids were added; however, he continued to have a chronic productive cough. A positron emission tomography scan was performed which showed fluorodeoxyglucose uptake of >10 standardized uptake value (SUV) in the left lower lobe consolidation with suspicion for malignancy versus atypical infection such as fungal or mycobacterial, despite negative cultures (Figure 2A). CT images showed worsening of the left lower lobe infiltrate (Figure 2B).

The patient was referred for a transbronchial cryobiopsy at our institution. Pathologic examination of the cryobiopsy specimens showed alveolar walls lined by cellular proliferation of columnar cells with abundant cytoplasm containing mucin, consistent with mucinous adenocarcinoma. Treatment was initiated with carboplatin, pemetrexed, and nivolumab. At the three-month follow-up, the patient was doing well and tolerating the current treatment.

## Discussion

Mucinous adenocarcinoma, as its name indicates, is a mucus-producing tumor [1]. It was formerly known as bronchioloalveolar cell carcinoma [3]. It accounts for about 5% of lung carcinomas [4]. The diagnosis can be difficult on small biopsy specimens [4]. This type of tumor does not have a specific clinical manifestation [3]. Mucinous adenocarcinomas are most commonly found in the lower lobe of the lungs, similar to our patient’s presentation [5].

Diagnosis of mucinous adenocarcinoma of the lung is usually delayed because its radiologic appearance mimics that of infectious pneumonia [6]. Moreover, this rare neoplasm has several forms. The pneumonic type shows a lobar consolidation on plain radiography and is particularly difficult to differentiate from pneumonia. However, CT imaging is helpful in diagnosing mucinous adenocarcinoma. Findings such as low-attenuating consolidation and the CT angiogram sign, which should show the enhanced pulmonary branches in the consolidation of the hypoattenuating parenchyma, after infusion of contrast medium may indicate mucinous adenocarcinoma [7], with cavitation formation in the consolidations being observed in 40% to 70% of cases [2]. The variant of mucinous adenocarcinoma with cavitation, such as in our patient, tends to have a worse prognosis than the noncavitary type [8].

Overall, mucinous adenocarcinomas are considered to have a prognosis and survival that is similar to that of non-mucinous adenocarcinomas. Moreover, the stage at diagnosis and molecular markers are important evolving areas in the evaluation and management of patients with lung adenocarcinoma [9]. The prognosis can be adversely or positively affected by the tumor’s genetic mutation status, especially when *EGFR *mutation is present [10]. Our patient’s genetic profile did not identify a targetable mutation; hence, he is currently being treated with carboplatin, pemetrexed, and nivolumab per the standard of care and tolerating this therapy well.

## Conclusions

We have presented a case of mucinous adenocarcinoma of the lung. This tumor is uncommon, difficult to distinguish on imaging from infectious pneumonia, and has a generally poor prognosis in the absence of targetable molecular mutations. Increased awareness of the disease presentation and the diagnostic challenges may help to improve the rates of diagnosis at early disease stages and improve outcomes. Clinicians should be aware of the unusual radiological manifestations of these rare lung tumors.
